# Supplementary figures and images for: Refined Mechanism of Mycoplasma mobile Gliding Based on Structure, ATPase Activity, and Sialic Acid Binding of Machinery
Source: mBio. 2019 Dec 24;10(6):e02846-19. doi: 10.1128/mBio.02846-19 (PMC6935860; doi:10.1128/mBio.02846-19)

**A**

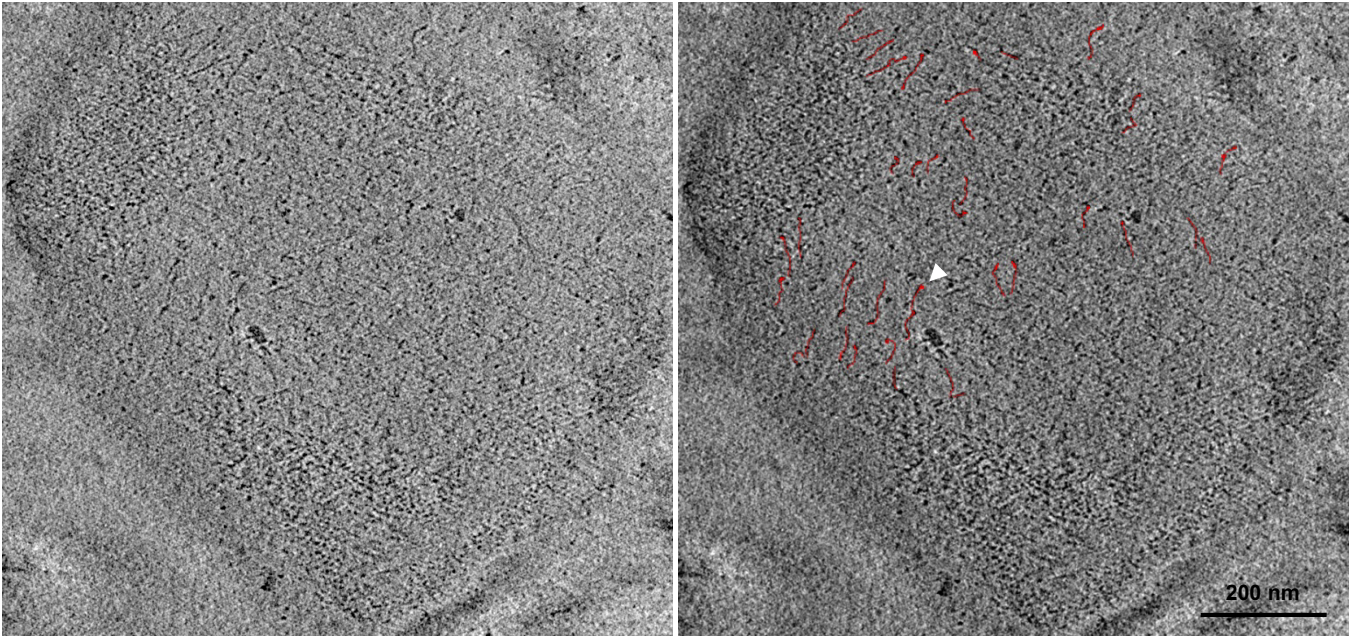

**B**

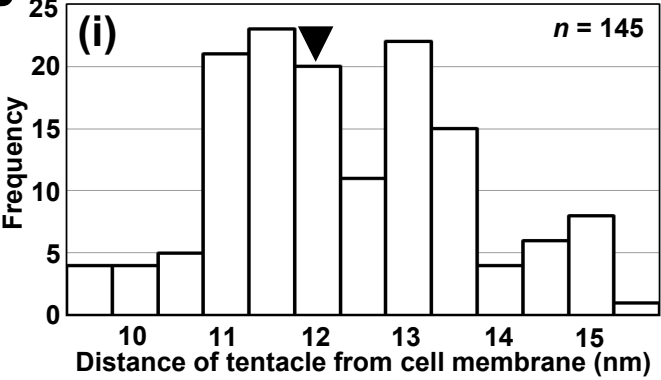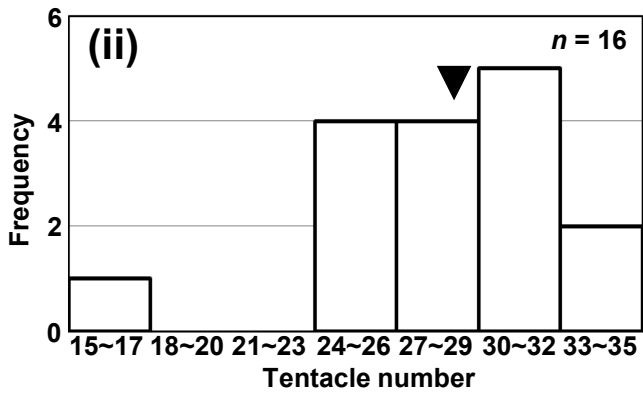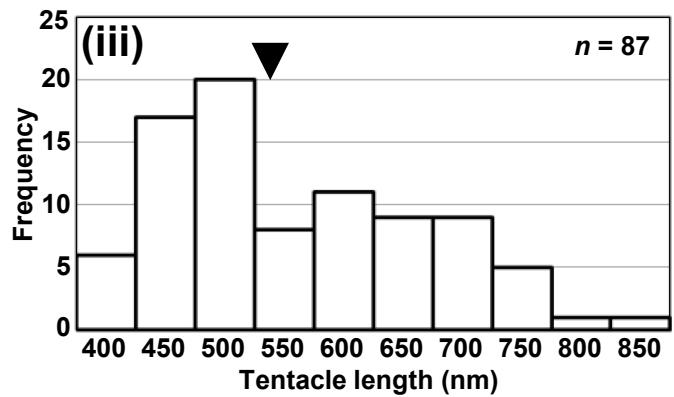

**FIG S1**

Supplement: FIG S1 [file mBio.02846-19-sf001.pdf]

**A**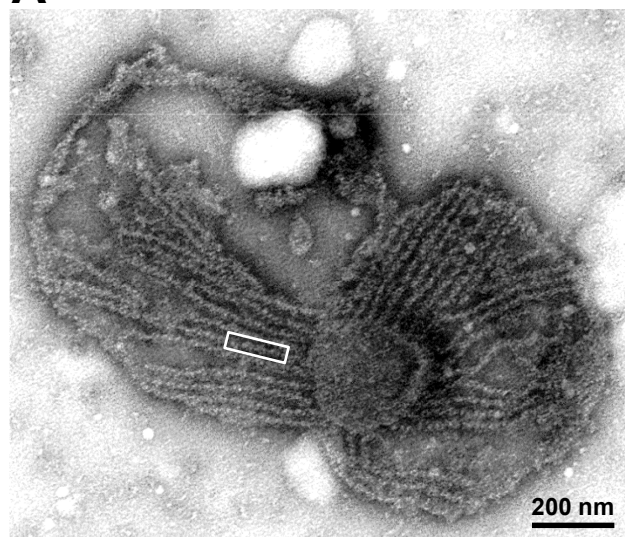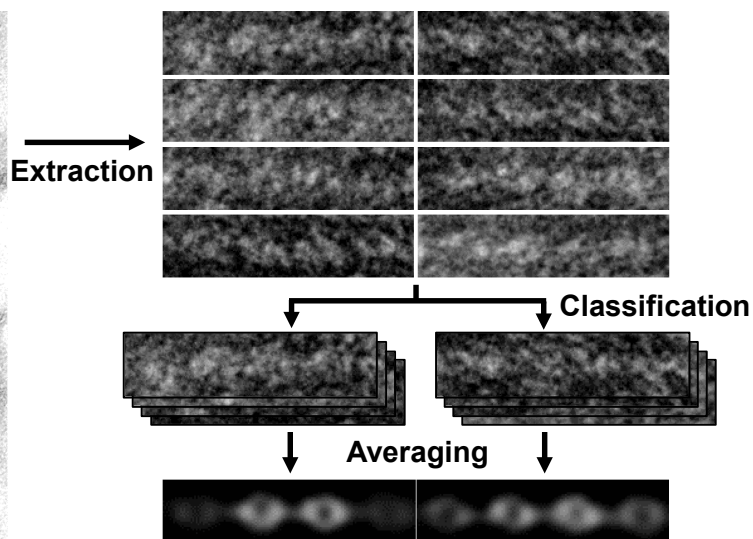**B**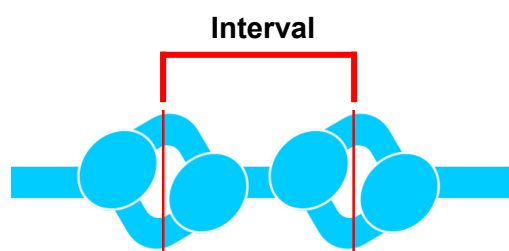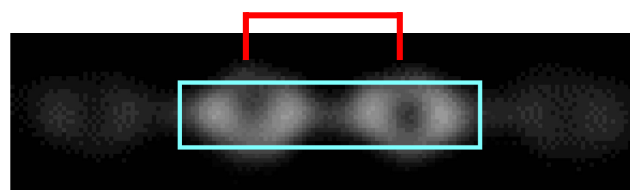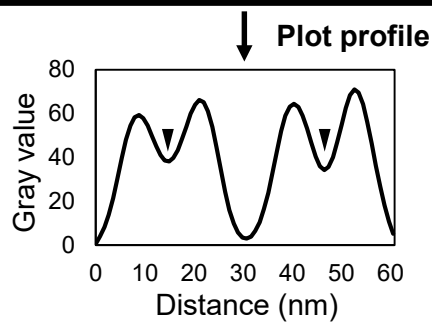**FIG S2**

Supplement: FIG S2 [file mBio.02846-19-sf002.pdf]

**A**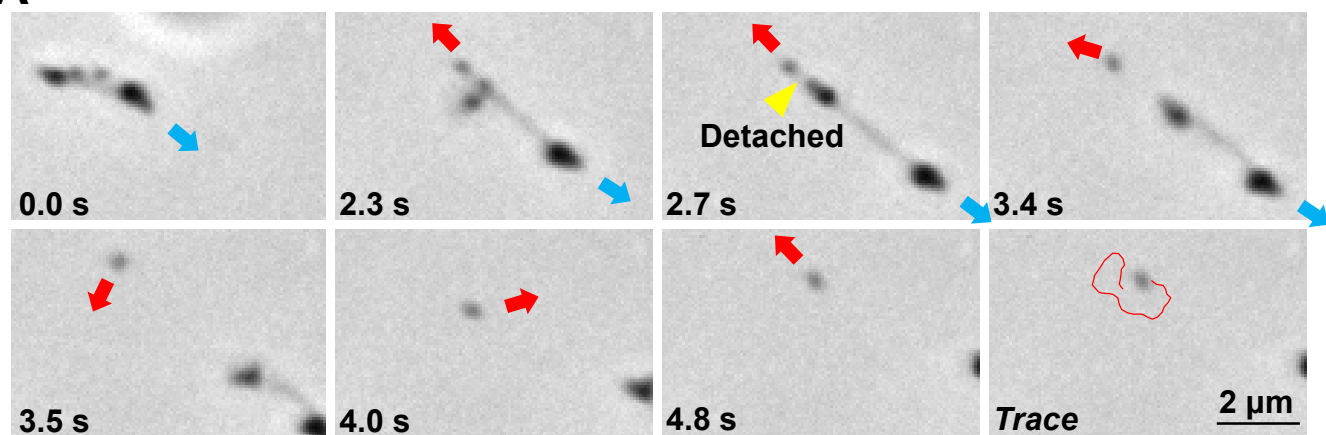**B**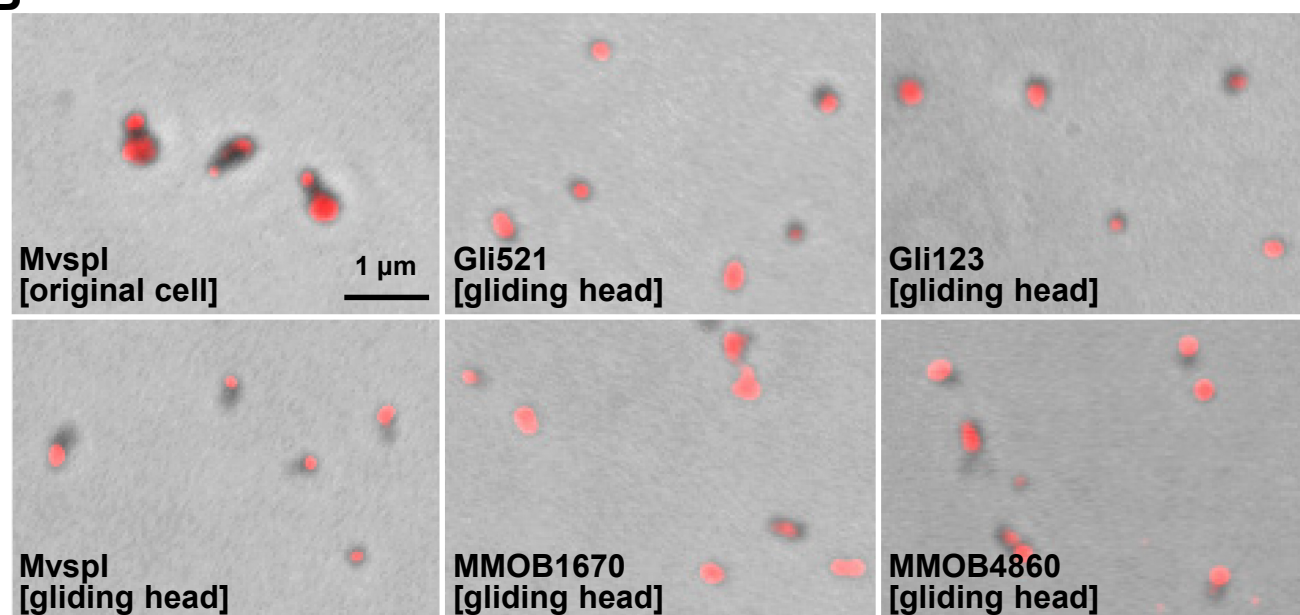**FIG S3**

Supplement: FIG S3 [file mBio.02846-19-sf003.pdf]

A

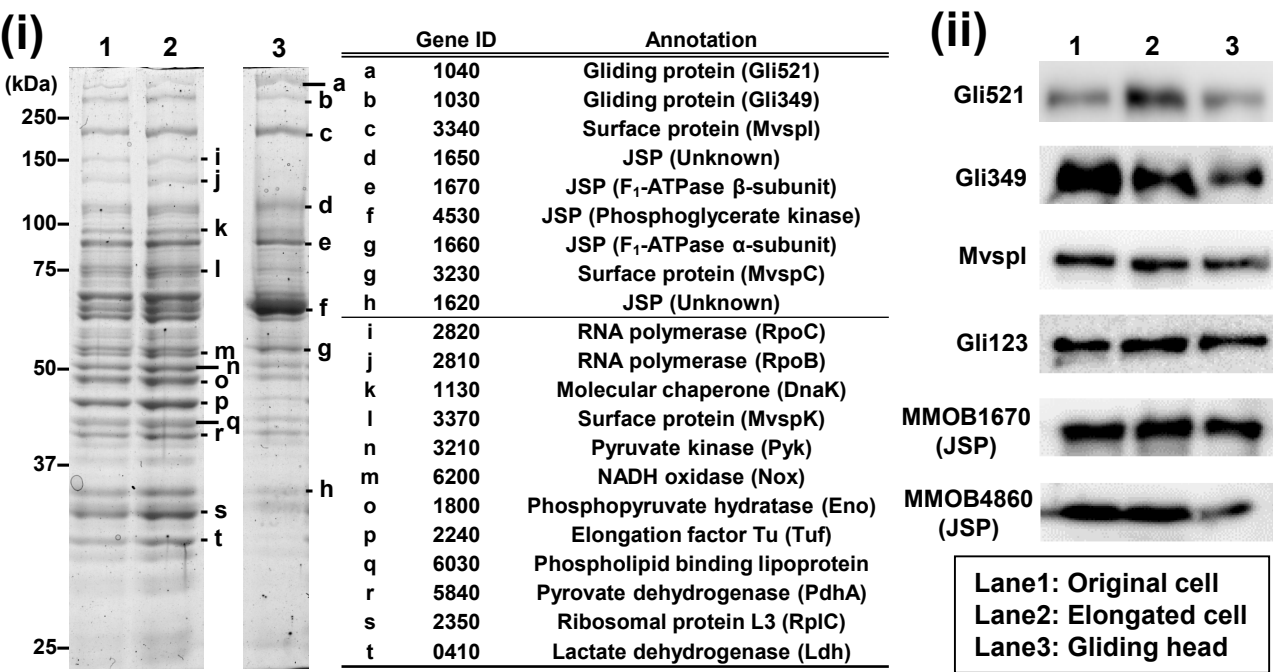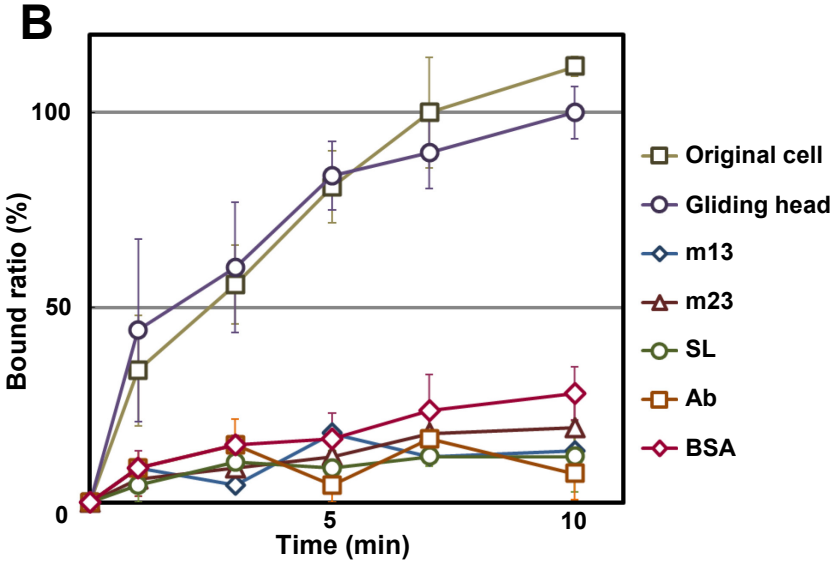

FIG S4

Supplement: FIG S4 [file mBio.02846-19-sf004.pdf]
